# An evaluation of the combination effect of zoledronate and chemotherapeutic agents in canine osteosarcoma cells

**DOI:** 10.3389/fvets.2024.1327377

**Published:** 2024-02-13

**Authors:** Yoshimi Iwaki, Stephanie E. S. Lindley, Noelle Bergman, Bruce F. Smith, Satyanarayana R. Pondugula

**Affiliations:** ^1^Department of Clinical Science, College of Veterinary Medicine, Auburn University, Auburn, AL, United States; ^2^Department of Veterinary Medicine and Surgery, College of Veterinary Medicine, University of Missouri, Columbia, MO, United States; ^3^Scott Ritchey Research Center, College of Veterinary Medicine, Auburn University, Auburn, AL, United States; ^4^Department of Anatomy, Physiology and Pharmacology, College of Veterinary Medicine, Auburn, AL, United States

**Keywords:** dogs, osteosarcoma, chemotherapy, zoledronate, combination effect

## Abstract

**Introduction:**

Osteosarcoma (OSA) is an aggressive form of bone cancer in both dogs and humans. The treatment options for metastatic (stage III) OSA are currently limited and the prognosis is poor. Zoledronate, a second generation amino-bisphosphonate, is commonly used for palliation of cancer induced bone pain. Zoledronate has also demonstrated anti-cancer properties and possibly enhances the cytotoxicity of doxorubicin in a canine histiocytosis cell line and human prostatic cancer cell line. The goal of this study was to evaluate the combination effect of zoledronate and various chemotherapeutic drugs in canine OSA cells.

**Methods:**

Canine OSA cell line (D17), cells from two canine primary OSAs, and MDCK, a canine kidney cell line, were used to evaluate the therapeutic potential of these drugs. Carboplatin, doxorubicin, vinorelbine, toceranib, and isophosphoramide mustard (active metabolite of ifosfamide) were used as chemotherapeutic agents. First, cells were treated with either zoledronate or chemotherapy drug alone for 72 hours. Cell viability was assessed using CellTiter Glo and IC_5_, IC_10_, IC_20_, and IC_50_ were calculated. Second, cells were treated with a combination of zoledronate and each chemotherapeutic agent at their IC_5_, IC_10_, IC_20_, and IC_50_ concentrations. After 72 hours, cell viability was assessed by CellTiter Glo.

**Results and discussion:**

Zoledronate, carboplatin, doxorubicin, vinorelbine, and isophosphoramide mustard showed concentration dependent decrease in cell viability. Toceranib showed decreased cell viability only at higher concentrations. When zoledronate was used in combination with chemotherapy drugs, while it showed potential synergistic effects with toceranib, potential antagonistic effects with vinorelbine and isophosphoramide mustard were observed. However, the results differed by cell line and thus, further evaluation is warranted to understand the exact mechanism of action.

## 1 Introduction

Osteosarcoma (OSA) is the most common bone tumor in dogs (1). This tumor exhibits locally aggressive and highly metastatic behavior (2). The current standard of care for canine appendicular OSA consists of local therapy (i.e., amputation, surgical limb-spare, or stereotactic radiation therapy) combined with cytotoxic chemotherapy (i.e., carboplatin, cisplatin, and/or doxorubicin) resulting in a median survival time of 10–12 months (2). Despite adequate local tumor control, greater than 90% of dogs are expected to develop and die from distant metastatic disease (2). Options to treat metastatic disease remain limited. Cytotoxic chemotherapy appears minimally effective for macroscopic disease with doxorubicin, cisplatin, mitoxantrone, ifosfamide, or toceranib phosphate (Palladia) demonstrating a response rate of 0–12% (3–6). The high frequency of metastasis and the resistance to chemotherapy have led to a search for novel treatment strategies.

Zoledronate is a third-generation bisphosphonate used in human and veterinary medicine for the treatment of cancer-associated bone pain, hypercalcemia, Paget’s disease, and osteoporosis (7). When administered systemically, bisphosphonates concentrate within metabolically active portions of bone and serve to inhibit osteoclasts and bone resorption. Bisphosphonates appear well-tolerated in dogs, and adverse events are limited (7, 8). Bisphosphonates have more recently been investigated for their anti-neoplastic properties. Zoledronate concentrates within osteoclasts and inhibits farnesyl pyrophosphate synthase, an enzyme required for the prenylation of GTP-binding proteins. This results in cell cycle arrest and apoptosis (9). Zoledronate also appears to modulate the tumor microenvironment by activating γδ T cells (10), inhibiting matrix metalloproteinase activity (11), promoting macrophage differentiation toward an anti-tumoral M1 phenotype (11), and decreasing vascular endothelial growth factor secretion by OSA cells (12). *In vitro* and *in vivo* murine studies have shown that zoledronate reduces OSA-induced bone lysis, reduces primary tumor growth, and decreases pulmonary metastasis through its effects on OSA cell migration, adhesion, and invasiveness (12–14). A previous publication described four human patients with metastatic OSA who were treated with zoledronate after failing chemotherapy. All four patients experienced a marked improvement in progression-free survival when compared to historical controls (>19 months vs. 2 months, respectively) (15). In the mice model of canine OSA, zoledronate mitigated the extent of bone lysis in affected bones. However, the volume of metastatic tumors in the lungs increased in the group of mice that received zoledronate (16). Another study investigated the impact of zoledronate on stage III canine OSA patients and found that it did not extend the survival of these dogs with OSA (17).

*In vitro* synergistic anti-cancer effects of bisphosphonates and chemotherapy drugs, specifically zoledronate and doxorubicin, have been observed in canine malignant histiocytosis cells (18), human prostate cancer cells (19), and human breast cancer cells (20). In these studies, the combination of zoledronate and doxorubicin increased cell apoptosis, while the combination of zoledronate with lomustine (CCNU) and vincristine did not increase cell apoptosis. It was indicated that zoledronate increased intracellular accumulation of doxorubicin in canine malignant histiocytosis cells; however, further mechanisms of this phenomenon are still undetermined (18). Previously, a phase I study of zoledronate was conducted in humans with newly diagnosed metastatic OSA (21). This study demonstrated that zoledronate could safely be administered alongside conventional cytotoxic chemotherapy. Still, the synergistic anti-cancer effects of bisphosphonates and chemotherapy drugs from *in vivo* studies are controversial. When zoledronate was used with ifosfamide in a rat model, it showed a complete resolution of osteosarcoma vs. progressive disease in the control group (22). However, a phase III clinical trial in human OSA patients demonstrated no benefit when zoledronate was initiated after diagnosis along with surgery and chemotherapy (23, 24).

Although zoledronate is commonly used in dogs with OSA, studies evaluating the effects of zoledronate and cytotoxic chemotherapy in combination for the treatment of canine OSA are lacking. This study aimed to evaluate the effect of zoledronate when combined with traditional cytotoxic chemotherapy in canine OSA cell lines. We hypothesized that concurrent use of zoledronate and cytotoxic chemotherapy will increase OSA cell death.

## 2 Materials and methods

### 2.1 Cell lines, primary cells, and cell culture

Canine kidney cell line (MDCK), canine OSA cell line (D17), and primary OSA cells (Ronald and Walter) were used for this study. MDCK was a gift from Dr. Kyriakis (Auburn University). This cell line was confirmed as to cell origin by PCR tests (IDEXX BioAnalytics, Columbia, MO, United States). D17 was a gift from BFS, and species-specific PCR was previously performed to confirm these as canine cells. Two primary OSA cells were obtained from client-owned dogs at the time of amputation by BFS. Neither dog received chemotherapy prior to the amputation. These cells were cultured in DMEM (Dulbecco’s Modified Eagle’s Medium, Corning) with penicillin (100 IU/mL, Corning), streptomycin (100 ug/mL, Corning), amphotericin B (0.5 ug/mL, Corning), and 10% FBS (fetal bovine serum, Sigma). The cell lines and primary OSA cells were maintained at 37°C (95% air, 5% CO2).

### 2.2 Zoledronate and chemotherapeutic agents

The chemotherapeutic agents, carboplatin (Sigma-Aldrich, St. Louis, MO, United States), doxorubicin (Sigma-Aldrich), vinorelbine (Sigma-Aldrich), toceranib (Sigma-Aldrich), isophosphoramide mustard (MedChemExpress, Monmouth Junction, NJ, United States), and the active metabolite of ifosfamide were used based on previous publications (2–6). Zoledronate (Sigma-Aldrich), carboplatin, and vinorelbine were dissolved in sterile distilled H2O at a final concentration of 10 mM. Doxorubicin and toceranib were dissolved in DMSO at a final concentration of 10 mM. Stock solutions were aliquoted and kept at −20°C for long-term storage. Isophosphoramide mustard was dissolved in DMSO at a final concentration of 100 mM. The stock solution of isophosphoramide was aliquoted and kept at −80°C. The stock solutions of carboplatin, doxorubicin, vinorelbine, and toceranib were utilized within 1 year after dissolution. Zoledronate and isophosphoramide mustard were used within 6 months following dissolution. Information on the stability of frozen solutions was only accessible for isophosphoramide mustard, and it remains stable within 6 months.

### 2.3 Evaluations of optimal doses of drugs

MDCK, D17, and primary OSA cells (Ronald and Walter) were suspended in cell culture medium and pipetted into a 96-well flat-bottomed plate using a final volume of 100 μL/well to give a final number of 5,000 cells/well. The cells were incubated for 24 h. Then, the cells were treated with chemotherapeutics alone or zoledronate alone. Chemotherapy agents and zoledronate were diluted in a cell culture medium before adding the intended concentration of each compound to the cells. Cells were incubated with chemotherapeutic drugs or zoledronate for 72 h. Control cells were incubated with a cell culture medium, and a vehicle was used to dissolve the drugs for 72 h. Puromycin (100 ng/mL) was used as a positive control for cytotoxicity. After the treatments, viability was determined by using a CellTiter-Glo cell viability Assay kit (Promega, Madison, WI, United States). CellTiter-Glo reagent (70 μL) was added to each well, and the plate was placed at room temperature for 15 min to stabilize the luminescence signal. The luminescence signal was measured using a luminometer (BMG LABTECH Inc., Cary, NC, United States). IC_5_, IC_10_, IC_20_, and IC_50_ were calculated by using GraphPad Prism (GraphPad Software, La Jolla, CA, United States). Cell viability was evaluated with triplicates and repeated 3 times for each concentration of each chemotherapy agent and zoledronate.

### 2.4 Evaluations of combinatorial effects

Cells were suspended and pipetted into a 96-well plate, as described above, and incubated for 24 h. Chemotherapy drugs and zoledronate were then added to the wells with the combinations of (a) no drug, (b) zoledronate alone with the concentrations of IC_5_, IC_10_, IC_20_, and IC_50_, (c) chemotherapy drug alone with the concentration of IC_5_, IC_10_, IC_20_, and IC_50_, and (d) chemotherapy drug plus zoledronate with each drug concentrations of IC_5_, IC_10_, IC_20_, and IC_50_. Cells were incubated at 37°C in a humidified atmosphere containing 5% CO_2_ for 72 h. Cell viability was determined by using the CellTiter-Glo cell viability Assay Kit as mentioned earlier. The luminescence signal was measured using the luminometer. Cell viability was evaluated with triplicates and repeated 3 times for each drug or combination of drugs. Combinational effects were evaluated based on figure appearances and *p*-values.

### 2.5 Statistical and data analysis

To compare cell viabilities in more than 2 groups, ANOVA followed by Tukey’s test was performed with GraphPad Prism 8. The data were normalized prior to analysis. Differences were considered statistically significant for *p*-values less than 0.05.

## 3 Results

### 3.1 Optimal dose finding

Zoledronate, carboplatin, doxorubicin, vinorelbine, and isophosphoramide mustard caused decreased cell viability in a dose-dependent fashion in the D17 cell line. Toceranib showed a significant reduction of cell viability above the concentration of 10 uM, and IC_5-50_ could not be calculated (Supplementary Figure 1; Supplementary Table 1). In primary OSA cells (Ronald), zoledronate, carboplatin, doxorubicin, and vinorelbine caused decreased cell viability in a dose-dependent fashion. Toceranib and isophosphoramide mustard showed increased cell viability at lower doses and decreased cell viability at higher doses (Supplementary Figure 2; Supplementary Table 2). IC_50_ of toceranib could not be calculated. In primary OSA cells from a different dog (Walter), zoledronate, carboplatin, doxorubicin, vinorelbine, and isophosphoramide mustard caused decreased cell viability in a dose-dependent fashion. Toceranib showed increased cell viability at lower doses and a significant reduction of cell viability above the concentration of 10 uM. IC_50_ of toceranib could not be calculated (Supplementary Figure 3; Supplementary Table 3). In MDCK cell line, zoledronate, carboplatin, doxorubicin, vinorelbine, toceranib, and isophosphoramide mustard caused decreased cell viability in a dose-dependent fashion (Supplementary Figure 4; Supplementary Table 4).

### 3.2 Evaluation of combinatorial effects

**Figure 1:**
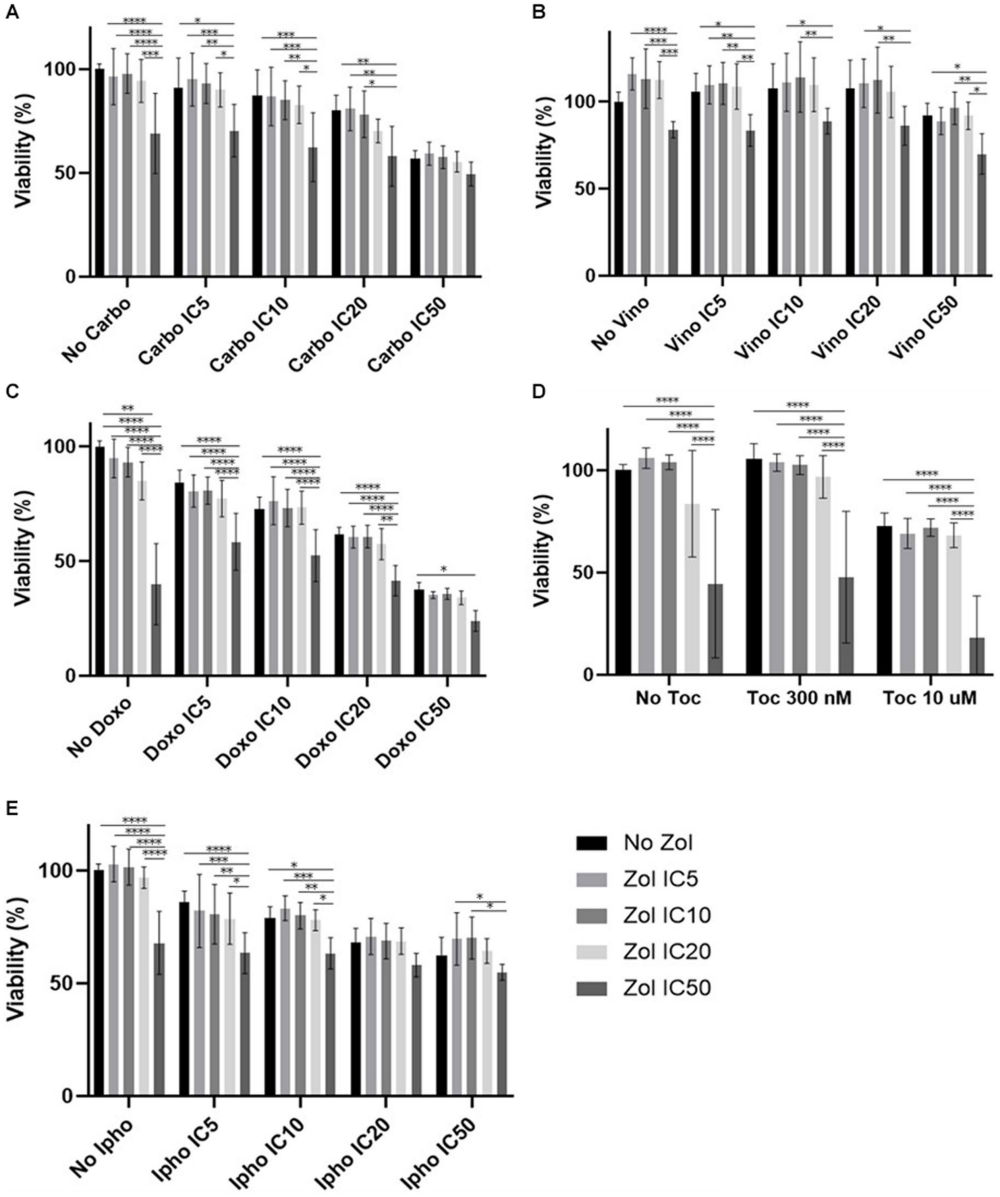
Cell viability of D17 cells treated with a combination of zoledronate and chemotherapy drugs. **(A)** Carboplatin, **(B)** Vinorelbine, **(C)** Doxorubicin, **(D)** Toceranib, **(E)** Isophosphoramide mustard. Data expressed as mean +/− SD and significance defined as ^*^p < 0.05, ^**^p < 0.01, ^***^p < 0.001, ^****^p < 0.0001.

**Figure 2:**
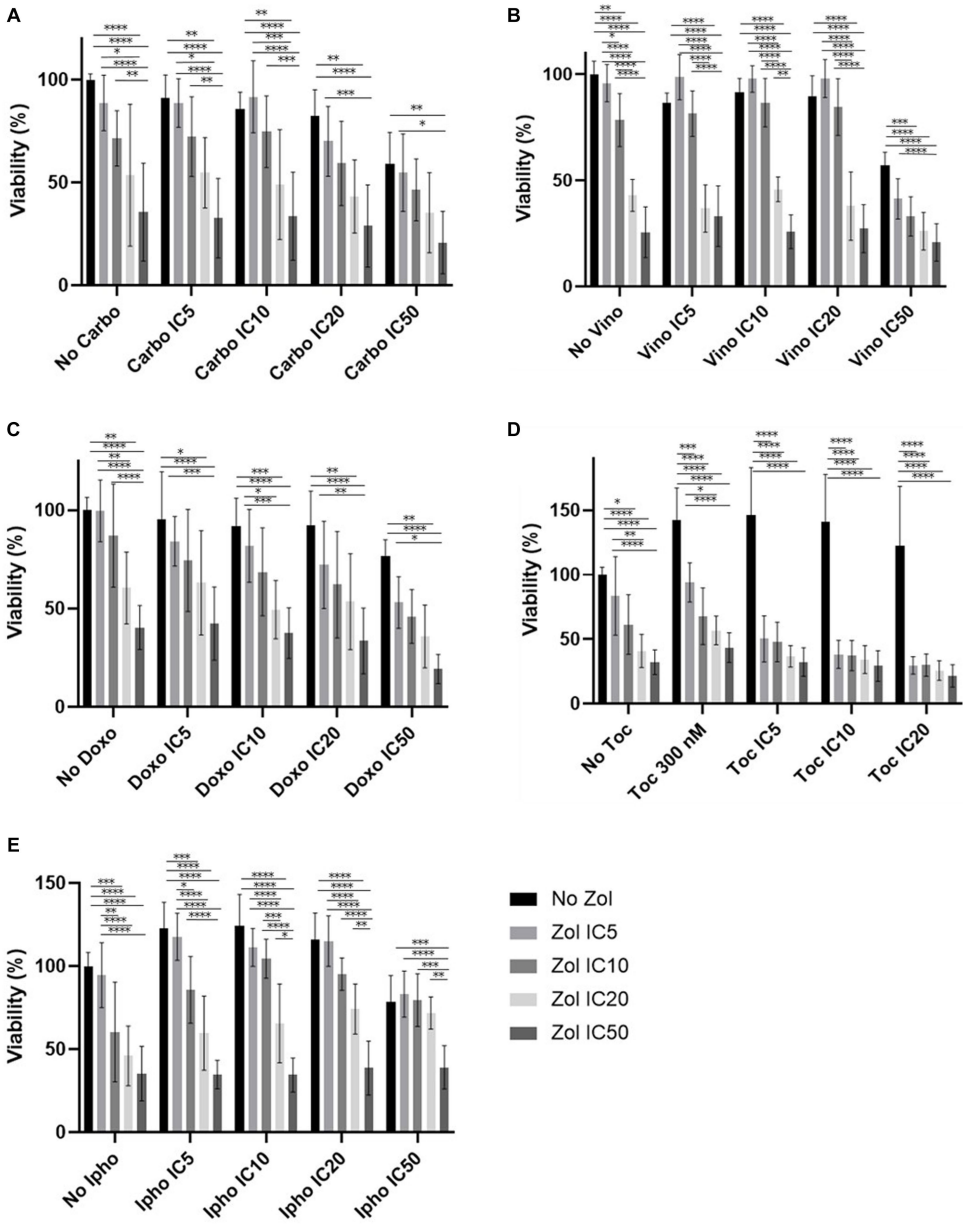
Cell viability of primary osteosarcoma 1 (Ronald), treated with a combination of zoledronate and chemotherapy drugs. **(A)** Carboplatin, **(B)** Vinorelbine, **(C)** Doxorubicin, **(D)** Toceranib, **(E)** Isophosphoramide mustard. Data expressed as mean +/− SD and significance defined as ^*^p < 0.05, ^**^p < 0.01, ^***^p < 0.001, ^****^p < 0.0001.

**Figure 3:**
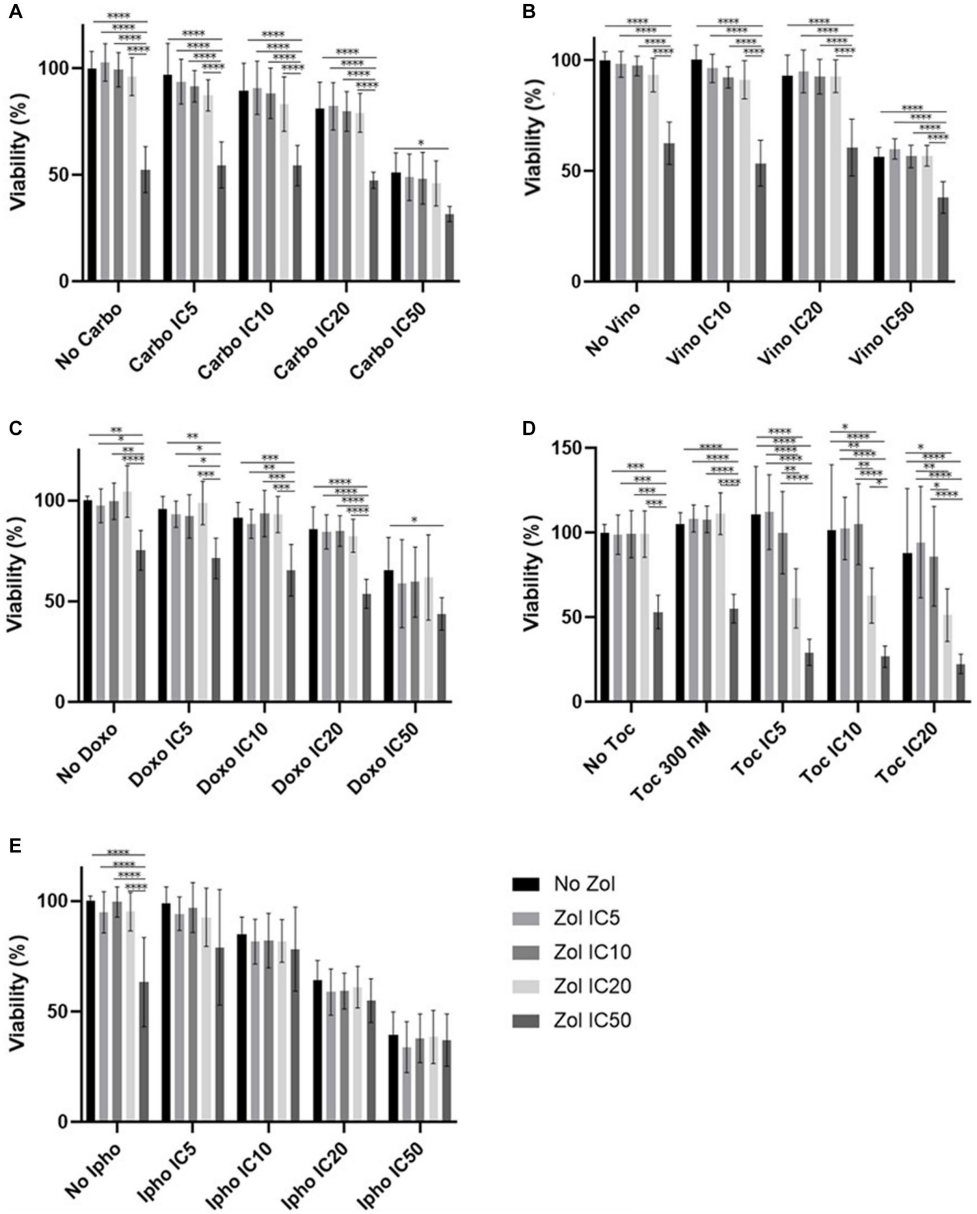
Cell viability of primary osteosarcoma 2 (Walter), treated with a combination of zoledronate and chemotherapy drugs. **(A)** Carboplatin, **(B)** Vinorelbine, **(C)** Doxorubicin, **(D)** Toceranib, **(E)** Isophosphoramide mustard. Data expressed as mean +/− SD and significance defined as ^*^p < 0.05, ^**^p < 0.01, ^***^p < 0.001, ^****^p < 0.0001.

**Figure 4:**
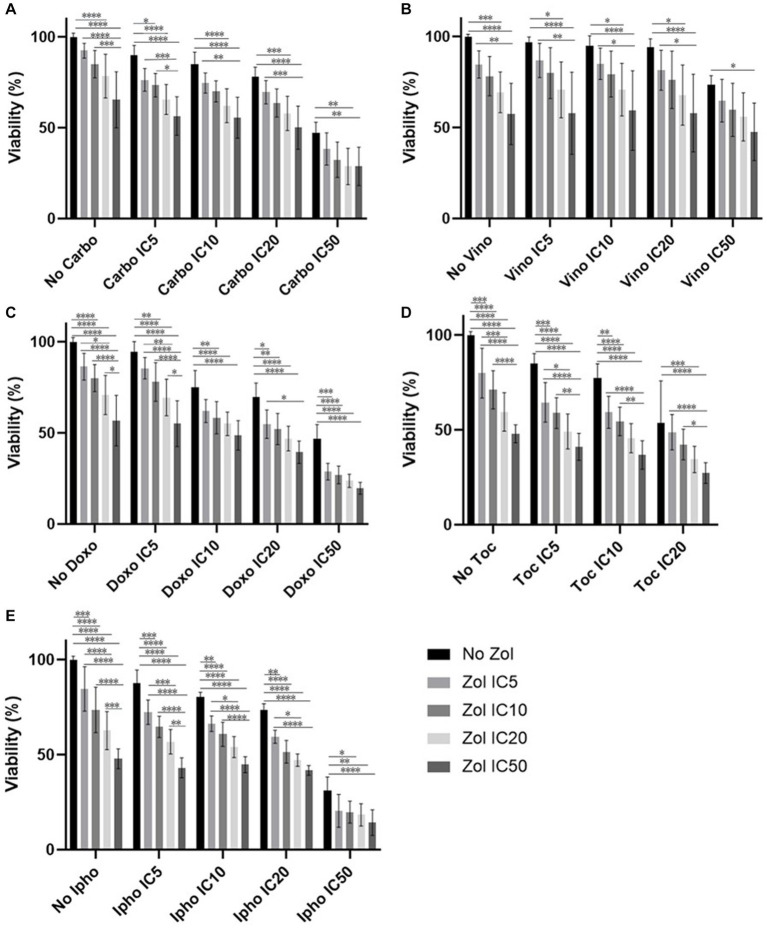
Cell viability of MDCK cell, treated with a combination of zoledronate and chemotherapy drugs. **(A)** Carboplatin, **(B)** Vinorelbine, **(C)** Doxorubicin, **(D)** Toceranib, **(E)** Isophosphoramide mustard. Data expressed as mean +/− SD and significance defined as ^*^p < 0.05, ^**^p < 0.01, ^***^p < 0.001, ^****^p < 0.0001.

Combinatorial effects were evaluated using IC_5_, IC_10_, IC_20_, and IC_50_, which were calculated (Supplementary Tables 1–4). In D17 cells, these values with toceranib could not be calculated. As a result, the concentration of 300 nM was used since that concentration was reported as Cmax in pharmacokinetics studies (25–31) (Supplementary Table 5). In addition, 10 uM of toceranib caused approximately 10% cell death based on the cell viability curve (Supplementary Figure 1) and this concentration was also used for combinatorial effect evaluation. Cell viability results with a combination of zoledronate and chemotherapy agents are shown in Figures 1–4. In D17 cells, there was no significant change in cell viability with the combination of carboplatin, doxorubicin, and toceranib. However, there was a tendency for antagonistic effects with vinorelbine and isophosphoramide (Figure 1). In primary OSA cell 1 (Ronald), there was no effect with carboplatin, doxorubicin, and isophosphoramide. There was a synergistic effect with toceranib. A tendency of antagonistic effects was observed in vinorelbine when combined with lower concentrations of zoledronate (Figure 2). In primary OSA cell 2 (Walter), there was no effect with carboplatin, vinorelbine, doxorubicin, and isophosphoramide. There was a tendency for a synergistic effect with toceranib when zoledronate was used in higher concentrations (Figure 3). In MDCK cells, no effects were noted with all chemotherapy drugs (Figure 4).

## 4 Discussion

The aim of this study was to evaluate the potential of zoledronate to enhance the therapeutic efficacy of cytotoxic chemotherapeutic agents in canine OSA cells. At optimal dose-finding, zoledronate, carboplatin, vinorelbine, doxorubicin, and isophosphoramide treatment alone resulted in a dose-dependent decrease in cell viability. In contrast, toceranib caused increased cell viability at lower concentrations and then showed a significant decrease in cell viability at higher concentrations. This non-monotonic effect of toceranib could be due to its unique mechanism of action. While carboplatin, vinorelbine, doxorubicin, and ifosfamide interact with DNA or the cell cycle directly and cause cell death, toceranib is a tyrosine kinase inhibitor and blocks receptor tyrosine kinases expressed on the cell surface by acting as a competitive inhibitor of adenosine triphosphate binding (32). The primary targets of toceranib are KIT, vascular endothelial growth factor receptor 2 (VEGFR2), platelet-derived growth factor receptor β (PDGFRβ), and FMS-like tyrosine kinases-3 (Flt-3) (32–34). Toceranib can target tumor cells by inhibiting receptor tyrosine kinases expressed on their surface that promote cell division and survival. For dogs, plasma VEGF concentration was associated with Cmax of toceranib and its minimum effective dose has been reported as 2.4 mg/kg (35). This information supports that toceranib might have a threshold value of its effectiveness. Due to its nature of competitive inhibition, toceranib might require a higher concentration to cause cell death. In the authors’ opinion, if the higher concentration is not achieved and toceranib only binds part of the targets, it might enhance opposite effects, such as enhancing cell signaling pathway or upregulation of cell surface receptors, which would favor increased viability.

In previous studies, zoledronate was shown to increase the cytotoxicity of doxorubicin in canine malignant histiocytosis cells (18), human prostate cancer cells (19), and human breast cancer cells (20). Our study is the first study that evaluated the effects of zoledronate in canine OSA cells when combined with several chemotherapy drugs. In this study, it was indicated that zoledronate may have a synergistic effect when used with toceranib. In addition, zoledronate may have antagonistic effects when used with vinorelbine and ifosfamide. Multiple mechanisms for the anti-neoplastic effect of zoledronate have been suggested, including increased intracellular accumulation of chemotherapeutics. Specifically, zoledronate caused increased intracellular doxorubicin accumulation in canine malignant histiocytosis cells (18). The mechanism for increased drug accumulation is unknown. The proposed mechanism includes altered intracellular drug metabolism via zoledronate-induced GTPase inhibition and decreased drug efflux via p-glycoprotein downregulation. While doxorubicin and vinorelbine are both drugs that efflux from cells via p-glycoprotein transporters, we found that doxorubicin and vinorelbine had different effects when they were combined with zoledronate, suggesting p-glycoprotein augmentation is not responsible for the drug increased activity. Based on this, other mechanisms likely contribute to the antagonistic effect of zoledronate with vinorelbine. As a next step, proteomics might help to determine which proteins are related to this phenomenon.

In human medicine, phase III randomized studies were performed in patients with OSA (23, 24). In these studies, OSA patients received chemotherapy or chemotherapy plus zoledronate pre- and post-surgery. The addition of zoledronate did not demonstrate a survival benefit and the zoledronate group developed additional adverse events including hypocalcemia and hypophosphatemia in comparison to the control groups. The investigators concluded that zoledronate should not be added to the standard OSA treatment. In these studies, they used two combination chemotherapy protocols—(1) methotrexate, etoposide, and ifosfamide or (2) doxorubicin, ifosfamide, and cisplatin. Based on our study, isophosphoramide mustard showed potential antagonistic effects, and this result supports the clinical outcomes reported in this phase III human OSA study.

Although side effects of zoledronate are not common, osteonecrosis of the jaw and elevation of BUN have been reported in dogs (8, 36). To evaluate any possibility of significant toxicity with the combinations of zoledronate of chemotherapeutic drugs, we used MDCK cells and analyzed their viability. In MDCK cells, there were no obvious synergistic or antagonistic effects from the combination of zoledronate and chemotherapeutic drugs; however, the cell viability seemed to decrease compared to D17 cells and primary OSA cells. This may indicate that dogs can have an increased risk of renal toxicity when zoledronate and chemotherapeutic drugs are used at the same time.

This was an *in vitro* study, and some conditions are different from *in vivo*. *In vivo*, cancer can create a tumor microenvironment that contributes to the further proliferation of cancer cells. There is an immunosuppressive barrier that helps cancer cells avoid immune destruction and includes regulatory T cells, myeloid-derived suppressor cells, mesenchymal stem cells, transforming growth factor-β, and immunoglobulins (37–39). Moreover, tumor macrophages and cancer-associated fibroblasts can promote cellular proliferation, invasion, and neo-angiogenesis (40, 41). *In vitro* studies lack these components, and it is difficult to completely imitate the true tumor reaction to these drugs that may be influenced by the microenvironment.

In this study, we used IC_5-50_ to evaluate the combination effects of drugs, and we did not use lower concentrations that did not cause cell death nor higher concentrations that caused increased cell death. This allowed us to evaluate the range of cell viability when we combined 2 drugs. The drug concentrations that we calculated in this study were slightly different from what has been reported in the *in vivo* pharmacokinetics studies (Supplementary Table 5); however, there was some overlap between reported drug concentrations in the canine body (25–31).

The other limitation of this study is that we did not evaluate the schedule dependency. Cell viability of canine OSA cells has been evaluated with the combination of radiation therapy and bisphosphonates (42). They evaluated the difference in cell viability when bisphosphonates are added either 24 h before, within 2 h, or 24 h after radiation therapy administration. In their study, they found that cell viability was significantly decreased when bisphosphonates were added 24 h after radiation. The mechanism is still unknown; however, changing the timing of bisphosphonate administration in relation to chemotherapy administration may be important for further evaluation.

In this study, each drug concentration resulted in different results; thus, the concentrations that can cause synergistic effects may or may not be achievable *in vivo*.

In conclusion, we found that zoledronate had synergistic effects with toceranib and antagonistic effects with vinorelbine and isophosphoramide. However, these effects were different depending on the cell line and concentration used, and further evaluation is needed to apply these treatments to clinical cases.

## Data availability statement

The original contributions presented in the study are included in the article/Supplementary material, further inquiries can be directed to the corresponding author.

## Ethics statement

Ethical approval was not required for the studies on animals in accordance with the local legislation and institutional requirements because only commercially available established cell lines were used.

## Author contributions

YI: Conceptualization, Formal analysis, Funding acquisition, Investigation, Methodology, Visualization, Writing – original draft. SL: Conceptualization, Funding acquisition, Writing – review & editing. NB: Conceptualization, Funding acquisition, Writing – review & editing. BS: Funding acquisition, Methodology, Resources, Supervision, Writing – review & editing. SP: Funding acquisition, Resources, Supervision, Validation, Writing – review & editing.
