# Supplementary material for: An evaluation of the combination effect of zoledronate and chemotherapeutic agents in canine osteosarcoma cells
Source: Front Vet Sci. 2024 Feb 13;11:1327377. doi: 10.3389/fvets.2024.1327377 (PMC10900512; doi:10.3389/fvets.2024.1327377)
Supplement: Supplementary file 1 [file Data_Sheet_1.docx]

Supplementary Material

Evaluation of the combination effect of zoledronate and chemotherapeutic agents in canine osteosarcoma cells

Yoshimi Iwaki*, Stephanie E.S. Lindley, Noelle Bergman, Bruce F. Smith, Satyanarayana R. Pondugula

*** Correspondence:** Yoshimi Iwaki: [yiktb@missouri.edu](mailto:yiktb@missouri.edu)


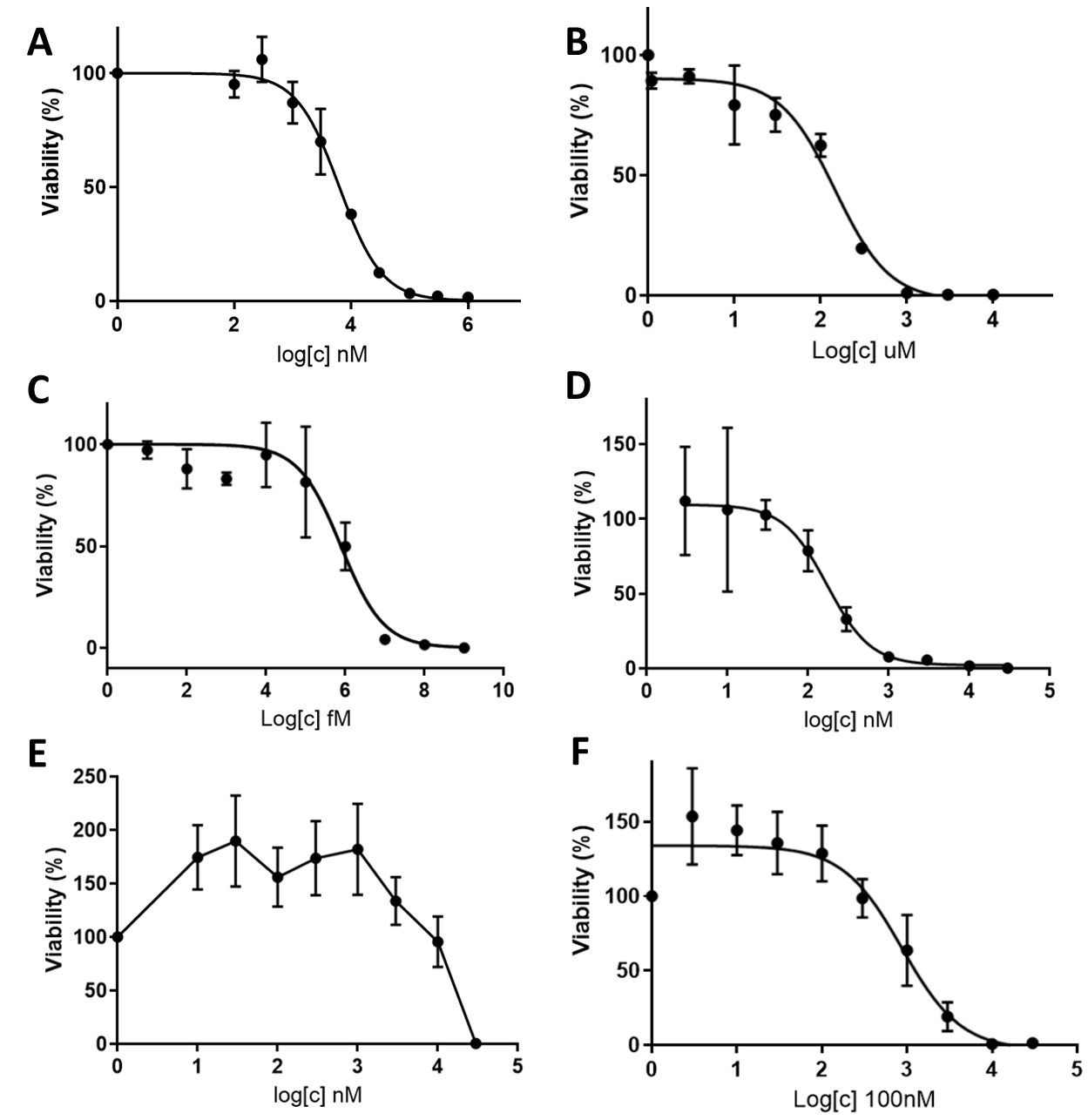
**Supplementary Figure 1.** Cell viability curve of D17 cells incubated with (A) zoledronate, (B) carboplatin, (C) vinorelbine, (D) doxorubicin, (E) toceranib, and (F) isophosphoramide mustard. Data expressed as mean +/- SD.

**Supplementary Table 1.** IC_5-50_ in D17 cell

|  | IC5 | IC10 | IC20 | IC50 |
| --- | --- | --- | --- | --- |
| Zoledronate | 547.5 nM | 1.00 uM | 1.95 uM | 6.04 uM |
| Carboplatin | 4.66 uM | 10.08 uM | 23.24 uM | 96.95 uM |
| Doxorubicin | 60.29 nM | 83.12 nM | 117.8 nM | 207.6 nM |
| Vinorelbine | 13.51 pM | 39.86 pM | 128.9 pM | 1.09 nM |
| Isophosphoramide | 34.2 uM | 48.7 uM | 71.4 uM | 86.9 uM |

**Supplementary Figure 2.** Cell viability curve of primary osteosarcoma cell-1 (Ronald), incubated with (A) zoledronate, (B) carboplatin, (C) vinorelbine, (D) doxorubicin, (E) toceranib, and (F) isophosphoramide mustard. Data expressed as mean +/- SD.


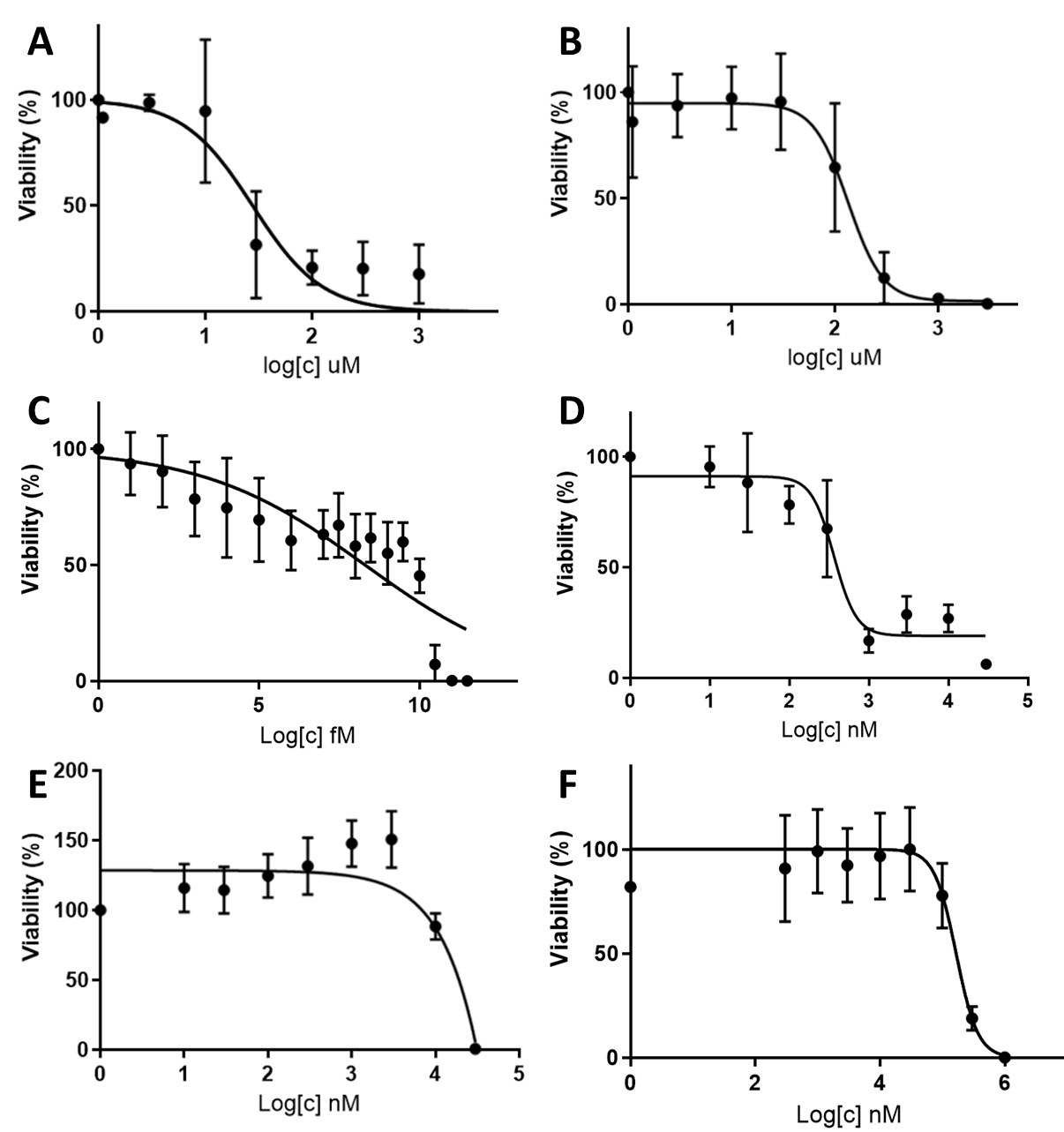


**Supplementary Table 2.** IC_5_-_50_ in primary osteosarcoma cell-1 (Ronald)

|  | IC5 | IC10 | IC20 | IC50 |
| --- | --- | --- | --- | --- |
| Zoledronate | 3.07 uM | 5.37 uM | 9.86 uM | 27.8 uM |
| Carboplatin | 35.08 uM | 48.84 uM | 69.92 uM | 129.2 uM |
| Doxorubicin | 10.43 nM | 26.22 nM | 71.33 nM | 394.9 nM |
| Vinorelbine | 7.76 fM | 589.7 fM | 64.8 pM | 199.9 nM |
| Toceranib | 8.82 uM | 9.77 uM | 10.92 uM | X |
| Isophosphoramide | 50.9 uM | 68.8 uM | 95.2 uM | 166.0 uM |

**Supplementary Figure 3.** Cell viability curve of primary osteosarcoma cell-2 (Walter), incubated with (A) zoledronate, (B) carboplatin, (C) vinorelbine, (D) doxorubicin, (E) toceranib, and (F) isophosphoramide mustard. Data expressed as mean +/- SD.


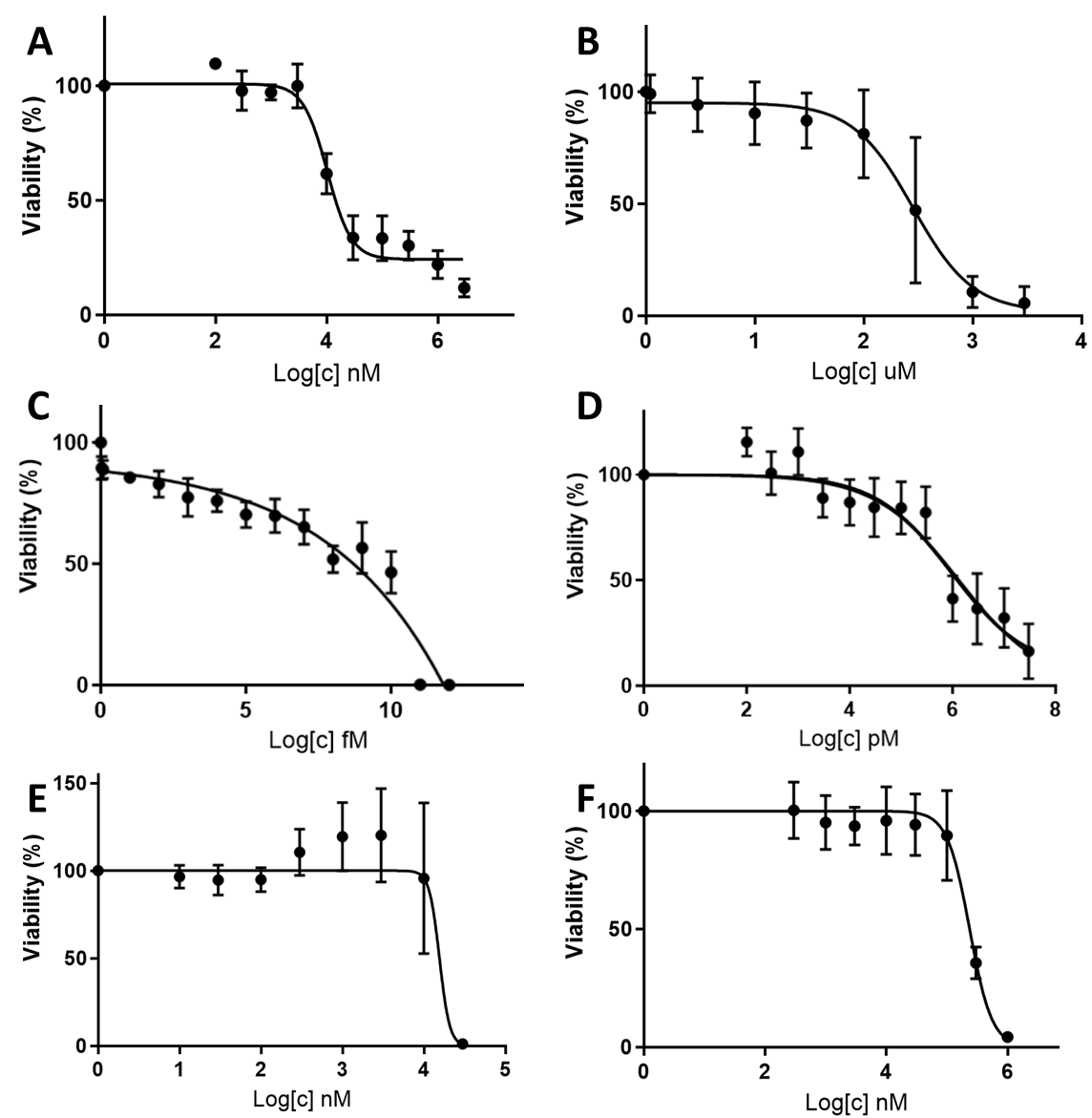


**Supplementary Table 3.** IC_5-50_ in primary osteosarcoma cell-2 (Walter)

|  | IC5 | IC10 | IC20 | IC50 |
| --- | --- | --- | --- | --- |
| Zoledronate | 270.3 nM | 726.4 nM | 2.12 uM | 13.29 uM |
| Carboplatin | 30.87 uM | 51.67 uM | 90.39 uM | 235.1 uM |
| Doxorubicin | 5.36 nM | 21.7 nM | 99.48 nM | 949.29 nM |
| Vinorelbine | X | 15.71 fM | 5.82 pM | 143.4 nM |
| Toceranib | 9.16 uM | 9.54 uM | 9.97 uM | X |
| Isophosphoramide | 70.29 uM | 94.39 uM | 129.97 uM | 224.56 uM |

**Supplementary Figure 4.** Cell viability curve of MDCK cell, incubated with (A) zoledronate, (B) carboplatin, (C) vinorelbine, (D) doxorubicin, (E) toceranib, and (F) isophosphoramide mustard. Data expressed as mean +/- SD.


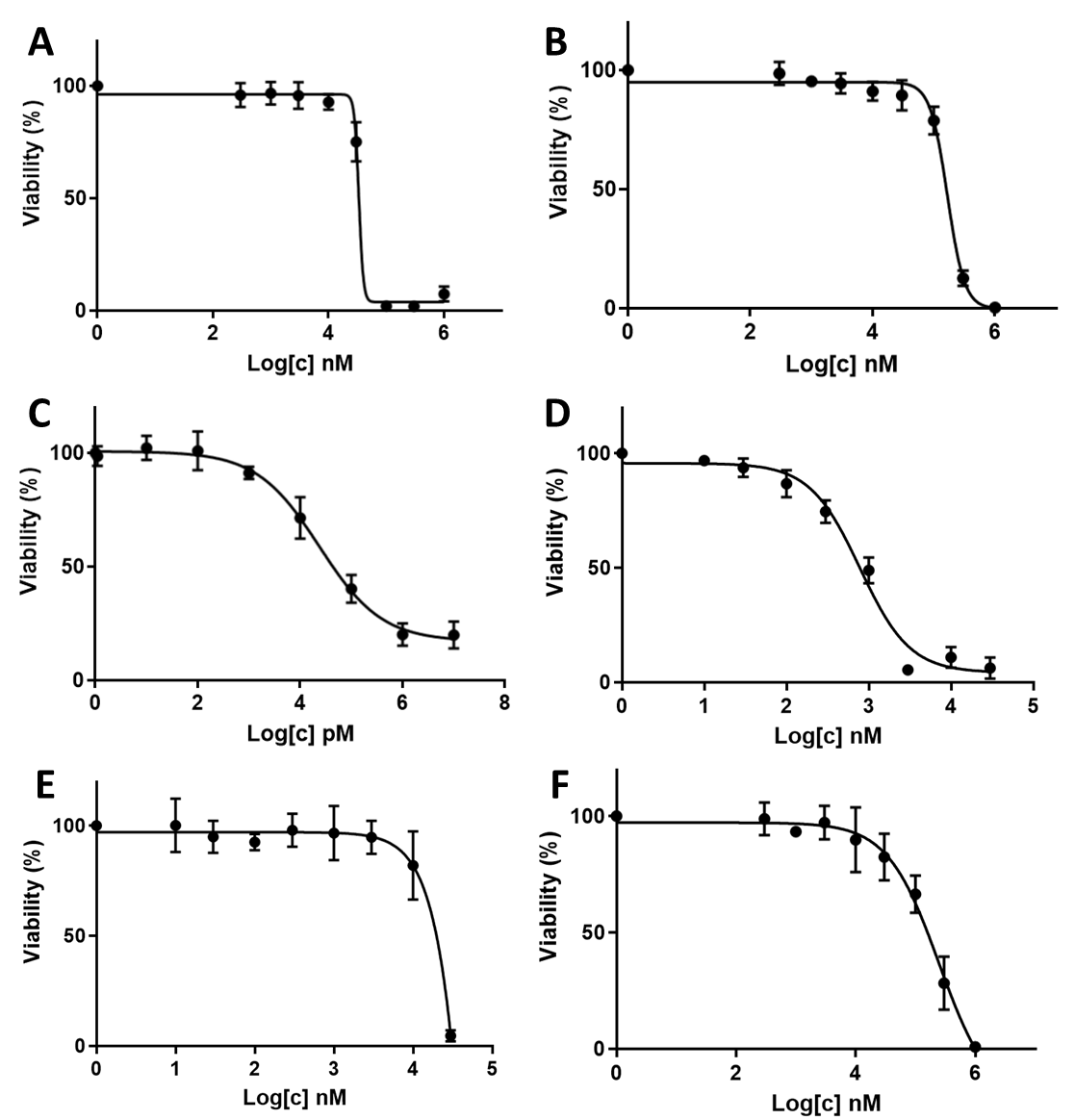


**Supplementary Table 4.** IC_5-50_ in MDCK cell

|  | IC5 | IC10 | IC20 | IC50 |
| --- | --- | --- | --- | --- |
| Zoledronate | 18.22 uM | 22.31 uM | 27.70 uM | 33.406 uM |
| Carboplatin | 54.90 uM | 71.52 uM | 95.31 uM | 165.69 uM |
| Doxorubicin | 55.38 nM | 107.3 nM | 220.1 nM | 781.5 nM |
| Vinorelbine | 140.4 pM | 673.1 pM | 3.68 nM | 23.73 nM |
| Toceranib | 7.03 uM | 8.44 uM | 10.29 uM | X |
| Isophosphoramide | 13.21 uM | 24.1 uM | 46.40 uM | 246.74 uM |

**Supplementary Table 5.** Cmax of drugs from the pharmacokinetics literatures

| Drug | Dose & administration | Cmax | Reference |
| --- | --- | --- | --- |
| Zoledronate | 0.25 mg/kg, IV, over 15 min | 4.4 uM (range 3.3-5.48 uM) | (1) |
| Carboplatin | 1.2 mg/kg, IV, over 20 min | Range 59.2 – 258.2 uM | (2) |
| Doxorubicin | 30 mg/m2, IV, over 30 min | 32.93 - 1067 ng/mL (range 15.3 nM - 1.83 uM) | (3, 4) |
| Vinorelbine | 0.4 mg/kg, IV | 56.4 - 98.3 ng/mL (range 52 - 91 nM) | (5) |
| Toceranib | 2.86 mg/kg, PO, every other day for 7 doses | Range 65 nM – 4.85 uM | (6) |
| Ifosfamide | 10 mg/kg | Range 17.2 - 47.5 uM | (7) |

Citations

1. Martin-Jimenez T, De Lorimier LP, Fan TM, Freise KJ. Pharmacokinetics and Pharmacodynamics of a Single Dose of Zoledronate in Healthy Dogs. *J Vet Pharmacol Ther* (2007) 30(5):492-5. doi: 10.1111/j.1365-2885.2007.00883.x.

2. Chen C, Wang W, Zhou H, Huang J, Liu P, Song T, Sun M. Pharmacokinetic Comparison between Systemic and Local Chemotherapy by Carboplatin in Dogs. *Reprod Sci* (2009) 16(11):1097-102. Epub 20090805. doi: 10.1177/1933719109341999.

3. Kobayashi S, Sakai T, Dalrymple PD, Wood SG, Chasseaud LF. Disposition of the Novel Anticancer Agent Vinorelbine Ditartrate Following Intravenous Administration in Mice, Rats and Dogs. *Arzneimittelforschung* (1993) 43(12):1367-77.

4. Selting KA, Ogilvie GK, Gustafson DL, Long ME, Lana SE, Walton JA, et al. Evaluation of the Effects of Dietary N-3 Fatty Acid Supplementation on the Pharmacokinetics of Doxorubicin in Dogs with Lymphoma. *Am J Vet Res* (2006) 67(1):145-51. doi: 10.2460/ajvr.67.1.145.

5. Kirsch M, Weisse C, Berent A, Clifford C, Leibman N, Wittenburg L, et al. Pilot Study Comparing Serum Chemotherapy Levels after Intra-Arterial and Intravenous Administration in Dogs with Naturally Occurring Urinary Tract Tumors. *Can J Vet Res* (2019) 83(3):187-96.

6. Yancey MF, Merritt DA, Lesman SP, Boucher JF, Michels GM. Pharmacokinetic Properties of Toceranib Phosphate (Palladia, Su11654), a Novel Tyrosine Kinase Inhibitor, in Laboratory Dogs and Dogs with Mast Cell Tumors. *J Vet Pharmacol Ther* (2010) 33(2):162-71. doi: 10.1111/j.1365-2885.2009.01133.x.

7. Germann N, Urien S, Rodgers AH, Ratterree M, Struck RF, Waud WR, et al. Comparative Preclinical Toxicology and Pharmacology of Isophosphoramide Mustard, the Active Metabolite of Ifosfamide. *Cancer Chemother Pharmacol* (2005) 55(2):143-51. Epub 20040914. doi: 10.1007/s00280-004-0894-y.
